# Recent Microdevice-Based Aptamer Sensors

**DOI:** 10.3390/mi9050202

**Published:** 2018-04-25

**Authors:** Donny Nugraha Mazaafrianto, Masatoshi Maeki, Akihiko Ishida, Hirofumi Tani, Manabu Tokeshi

**Affiliations:** 1 Graduate School of Chemical Sciences and Engineering, Hokkaido University, Kita 13 Nishi 8, Kita-ku, Sapporo 060-8628, Japan; donnymaza@eis.hokudai.ac.jp; 2 Division of Applied Chemistry, Faculty of Engineering, Hokkaido University, Kita 13 Nishi 8, Kita-ku, Sapporo 060-8628, Japan; m.maeki@eng.hokudai.ac.jp (M.M.); ishida-a@eng.hokudai.ac.jp (A.I.); tani@eng.hokudai.ac.jp (H.T.); 3 ImPACT Research Center for Advanced Nanobiodevices, Nagoya University, Furo-cho, Chikusa-ku, Nagoya 464-8603, Japan; 4 Innovative Research Center for Preventive Medical Engineering, Nagoya University, Furo-cho, Chikusa-ku, Nagoya 464-8601, Japan; 5 Institute of Innovation for Future Society, Nagoya University, Furo-cho, Chikusa-ku, Nagoya 464-8601, Japan

**Keywords:** microdevice, aptamer, biosensor, SELEX, lab-on-chip, point-of-care

## Abstract

Since the systematic evolution of ligands by exponential enrichment (SELEX) method was developed, aptamers have made significant contributions as bio-recognition sensors. Microdevice systems allow for low reagent consumption, high-throughput of samples, and disposability. Due to these advantages, there has been an increasing demand to develop microfluidic-based aptasensors for analytical technique applications. This review introduces the principal concepts of aptasensors and then presents some advanced applications of microdevice-based aptasensors on several platforms. Highly sensitive detection techniques, such as electrochemical and optical detection, have been integrated into lab-on-a-chip devices and researchers have moved towards the goal of establishing point-of-care diagnoses for target analyses.

## 1. Introduction

In the past decade, technologies for analytical detection sensors have undergone significant growth. Conventional sensors are robust, reliable, and provide high reproducibility of measurements. However, their main drawback is that they cannot be integrated into a compact packaging flow, which in many analysis cases is critical. Beyond this, expensive instrumentation and long analysis time are general problems to be considered. For these reasons, microdevice platforms offer an attractive alternative to conventional techniques [1]. Furthermore, microdevices are also important for reducing the amount of sample required, for alleviating interferences or cross-contamination by their disposable design, and for integrating multiple sensor arrays to increase the throughput. Sensors perform three functions: targeting an analyte, recognizing an element, and transducing a signal. The analyte interacts in a selective way with the recognition site, which shows some affinity or a catalytic reaction. In a biosensor, the recognition system is based on biochemical or biological sensing elements such as antibodies, enzymes, nucleic acids, or aptamers [2]. These elements are commonly immobilized on a physicochemical transducer and combined with a detector to generate an electronic signal readout that is proportional to the quantity of the target. Antibodies and enzymes have made a big contribution to a wide range of applications that are based on molecular recognition. The use of antibodies became widespread by the 1970s, when polyclonal techniques from immunized animals was a popular choice [3]. The catalytic enzyme-based recognition system is very attractive in biosensor applications due to a variety of measurable reaction products arising from the catalytic process, which includes protons, electrons, light, and heat. Despite the fact that antibodies and enzyme based assays are established as a standard method for analytical detection, they are still restricted in recognizing several small molecules or non-immunogenic targets, which are not easy to analyze and differentiate.

Oligonucleotides such as RNA, DNA or peptides can be used as the receptor for the recognition of specific small organic molecules or even as a complementary strand in the hybridization process. The name of such an oligonucleotide is aptamer (“aptus” meaning “fitted” and “meros” meaning “part”) [4]. Some aptamers contort into three-dimensional (3D) conformations that can bind to target molecules in stable complexes and they commonly rely on van der Waals forces, hydrogen bonds, or electrostatic interactions [5]. Aptamers play a role similar to antibodies. However, they are obtained by a chemical synthesis that is easily modified, more stable, and inexpensive. Also, aptamers can discriminate between highly similar molecules, such as theophylline and caffeine, which differ by only a methyl group [6]. In addition, after performing the recognition function, aptamers can be efficiently regenerated without loss of either sensitivity or selectivity [7]. All these features make aptamers very suitable as a receptor in bio-sensing applications than antibodies.

**Figure 1:**
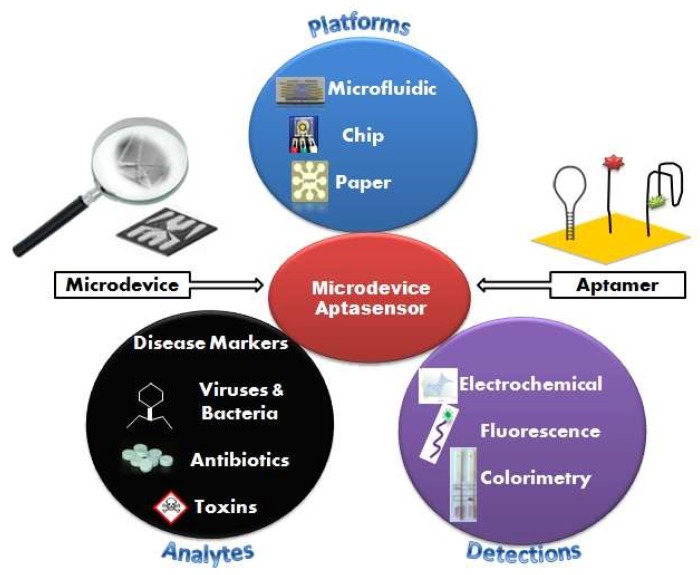
Schematic illustration of microdevice-based aptamer sensor with various platforms, detection methods and application to actual samples.

This review, as described in Figure 1, addresses the current state of research related to microdevice instruments and the advantage of emerging aptamer biosensor for numerous applications and target analysis. It is divided into three parts: (i) classification of microdevice platforms; (ii) detection methods and assay formats; and (iii) applications to actual samples. Current work in aptamer selection-based microdevices and characterizations are also covered, and future perspectives in the field are offered.

## 2. The SELEX Method (In-Vitro Selection)

Aptamers are oligonucleotides, commonly 12–80 nucleotides long, and they have a function to act as specific affinity receptors towards a broad spectrum of numerous targets including small organic molecules, proteins, cells, viruses, and bacteria. New aptamers are originated by an in vitro selection process known as the SELEX (Systematic Evolution of Ligands by EXponential enrichment) method. This method was simultaneously developed by Tuerk and Gold [8] and Ellington and Szostak [9], in 1990. The SELEX method contains several steps such as incubation, separation, amplification, and purification. Briefly, a library of randomized RNA or DNA sequences is incubated with the target of interest. The sequences with no affinity or only a weak affinity to the target are removed from the library, while the sequences that have strong binding are then recovered and amplified using a polymerase chain reaction (PCR), this process narrows down the aptamer candidates. The selection process is repeated approximately 7 to 15 times to create a sufficiently narrow pool of aptamer candidates that can then be characterized to determine their efficiency.

A conventional SELEX method requires extensive manual handling of reagents, and it is time-consuming, typically requiring a dozen or more rounds of repeating the method and weeks to months to achieve suitable affinity. The integration of several SELEX steps in a single small platform is an appealing trend in the field. It offers a range of capabilities of high-resolution separation between oligonucleotide candidates using small quantities of reagents and samples. A single-round screening of aptamers was reported and this marked the innovation of a fully automated and integrated miniaturized SELEX process [10].

## 3. Classification of Microdevices

### 3.1. Microfludic Devices

Microfluidics, also known as “lab-on-a-chip,” is an emerging technology that represents a revolution in laboratory experimentation, bringing the benefits of integration, miniaturization, and automation to many research areas. It is the science and technology of systems that control small amounts (10^−9^–10^−18^ L) of fluids in channels with dimensions of submillimeter to submicrometer [11]. The reduced dimensions and volumes in microfluidic channels allow all tasks to be done with much less sample than what otherwise might be used. It is beneficial to improve the transport of analyte from the sample volume to the biorecognition element, in particular for a surface-bound sensing element [12]. In recent years, the development of microfluidic chips as a miniaturized diagnostic platform has attracted the attention of researchers. The basic operating units of biochemistry analysis, e.g., sample preparation, reaction, and separation tests, can be integrated into a micron scale chip, and then the whole analysis process can be completed automatically.

#### 3.1.1. Microfluidic SELEX Devices

**Figure 2:**
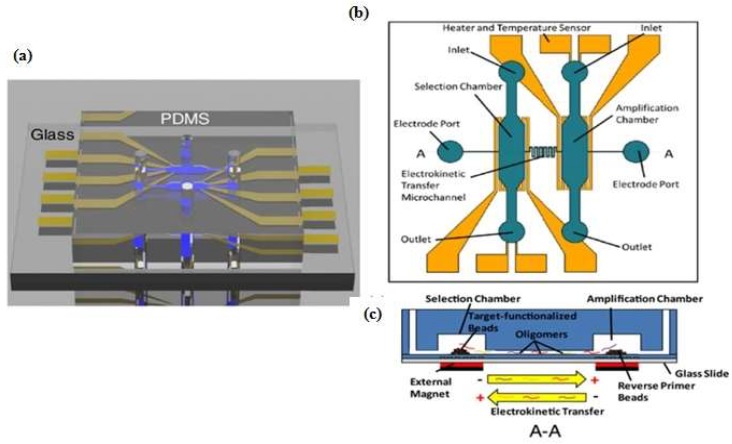
Schematic of microfluidic SELEX device which integrates selection and amplification steps. (**a**) Polydimethylsiloxane (PDMS) channel on glass substrate; (**b**) Top view with detailed features; (**c**) Selection and amplification microchamber connected by a single serpentine shaped microchannel. Reproduced with permission from reference [17]. Copyright 2017 Electrochemical Society.

**Figure 3:**
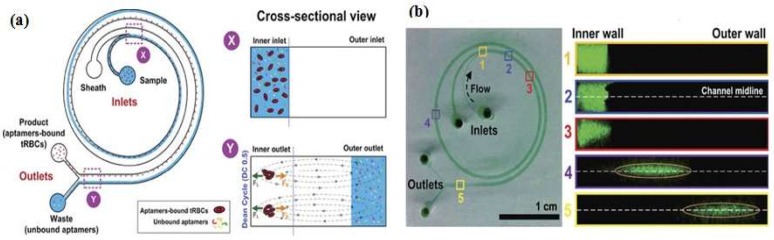
Bi-loop spiral design of inertial microfluidic SELEX (I-SELEX) with dual inlets and outlets. (**a**) The unbound oligonucleotide/any particles migrate towards the outer-side wall (blue color) and are separated with the desired target; (**b**) Numbers 1–5 represent cross sections inside the channel. Fluorescence-labeled aptamer was used to identify each position. Reproduced with permission from reference [19]. Copyright 2015 Macmillan.

One example that combines the advantages of the SELEX method and microfluidic systems into a compact platform design is a competitive assay test of the selected aptamer to reduce the number of sequences subjected to sequencing and affinity characterization. The entire SELEX process is shortened and the possibility to produce the aptamer as a biorecognition element is increased [13]. Integration of the affinity selection and amplification steps in SELEX by combining bead-based biochemical reactions has been demonstrated [14,15,16,17,18]. A simple microfluidic SELEX device was developed by Olsen et al. [17], this device was fabricated using single layer soft lithography (Figure 2). In this work, an electrokinetic microfluidic device for aptamer enrichment was demonstrated as an integrated microfluidic device without requiring an offline process. The electrokinetic microfluidic device features a microchamber and an electrokinetic transfer microchannel that allows oligonucleotide migration under an electric field. A heater and temperature sensor are used to control the target-aptamer binding and amplification process through PCR thermal cycling. In another example, Birch et al. [19] developed an inertia microfluidic SELEX or I-SELEX device to establish a system for continuous partitioning of cell-bound aptamers away from unbound nucleic acids in a bulk solution. The device was fabricated from polydimethylsiloxane (PDMS) and bonded to microscopic glass slides and had bi-loop spiral with double inlets-outlets (Figure 3). The working process began by pumping the target-aptamer library and buffer through each inlet, then the unbound aptamers migrate along the outer wall towards the waste outlet. Using this strategy, they successfully identified a high-affinity aptamer that was a subset of specific interactions with distinct epitopes on malaria-parasite infected red blood cells. In order to improve efficiency and selectivity, some groups have developed techniques such as the volume dilution challenge microfluidic SELEX (VDC-MSELEX) [20], dielectrophoresis and electrophoresis SELEX [21], SELEX assisted by graphene oxide (GO) [22], surface plasmon resonance (SPR)-based SELEX methods [23,24]. SPR-based SELEX methods have attracted attention in recent years because selection and evaluation can be performed simultaneously without labeling the sensor.

#### 3.1.2. Microfluidic Chip Aptasensors

**Figure 4:**
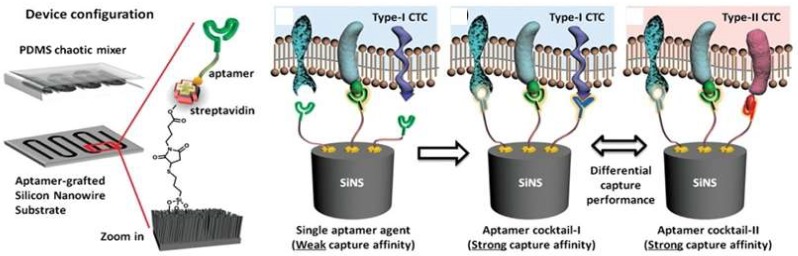
A representative chaotic mixer microfluidic device combined with an aptamer cocktail-grafted silicon nanowire substrate (SiNS). The different aptamers work synergistically to enhance capture affinity in a low-concentration target. Reproduced with permission from reference [68]. Copyright 2016 John Wiley and Son.

Microfluidic chips are a device or micro-channel that integrates a fluidic system including steps for transporting, mixing, preparing, and detecting a sample. Dimensions of the device must be in the range of a millimeter to a few square centimeters [25]. In recent years, microfluidic chips have aroused increasing interest for various application because of their desirable features, such as the smaller sample amount needed and lowered reagent consumption. The substrate materials of microfluidic chips such as polymers (e.g., PDMS, PMMA, PS) [26,27,28,29,30,31,32,33,34], ceramics (e.g., glass) [6,13,14,16,17,18,19,21,25,35,36,37,38,39,40,41,42,43,44,45,46,47,48,49,50,51,52,53,54,55,56,57,58,59,60,61,62,63], and semiconductors (e.g., silicon) [64,65,66,67,68,69,70,71,72,73], are currently used to obtain mechanical strength. Many researchers utilize PDMS and the soft lithography technique to fabricate microfluidic devices due to their easiness of use and simple process. Prototypes can be rapidly built and tested because researchers do not waste time with laborious fabrication protocols. Contrary to common beliefs, soft lithography does not require hundreds of square meters of clean room space. Indeed, a small bench space under a lab fume hood is sufficient for placing PDMS prototyping instruments to quickly assess a microfluidic technique. Recently, Ma et al. [62] developed a very attractive design for a volumetric bar chart chip (V-chip) aptasensor. This group applied a distance-readout method combined with aptamer-responsive hydrogel. Platinum nanoparticles (PtNPs) were used to encapsulate aptamer and hydrogel. Upon introduction of the target, the aptamer bound with the target then induced disruption of the hydrogel and released the PtNPs. Subsequently, the hydrogel was loaded into the volumetric bar chart chip while the PtNPs catalyzed the reaction of H_2_O_2_ to produce O_2_. The colored ink flow in the V-chip was triggered by O_2_ and was quantitatively related to the concentration of the target. Although the instrument design was very simple, it still needs to treat the sample with an immunoaffinity column similar to conventional methods. Zhao et al. [68] fabricated an aptamer-grafted silicon nanowire substrate (SiNS) embedded microfluidic chip and chaotic mixer PDMS for sensitive detection of circulating tumor cells (CTCs). As a cancer marker, the presence of CTCs in blood is very rare and it is difficult to repeatedly observe them during the treatment, so Zhao et al. developed an aptamer-cocktail form with a synergistic effect (two or more aptamers may work synergistically, this phenomenon leads to increased cell affinity) (Figure 4). They constructed the cell-SELEX to produce multiple aptamers that were immobilized on the microfluidic device. In order to ensure the synergistic effect, they switched the position and number of aptamers to examine optimal conditions. Furthermore, they also evaluated the cell capture efficiency as a function of aptamer density and found that the efficiency gradually increased with aptamer density.

**Figure 5:**
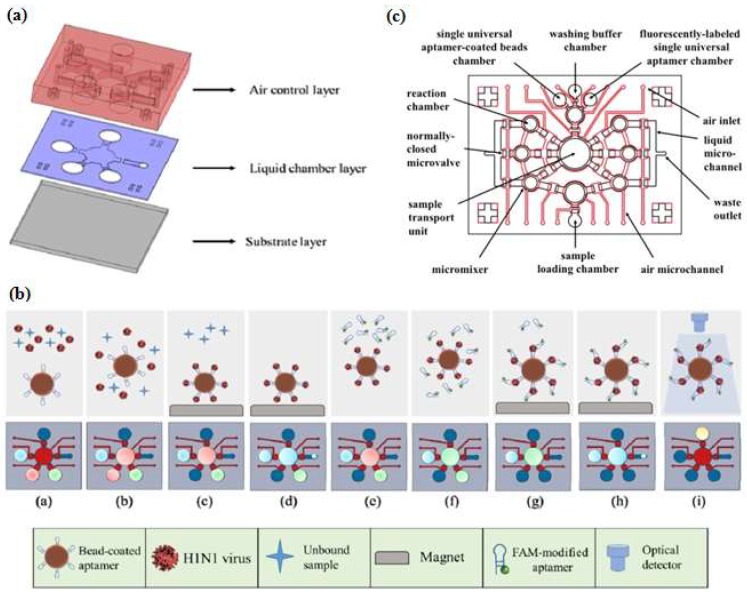
Integrated microfluidic chip system using a sandwich aptamer. (**a**) The device was composed of PDMS structures (air control layer & liquid chamber layer) and a glass substrate; (**b**) Schematic ilustration of experimental procedure performed on the integrated microfluidic chip system. Reproduced with permission from reference [47]. Copyright 2016 Elsevier. (**c**) The configuration of the inlet-outlet, chambers, micromixers, and microvalve. Reproduced with permission from reference [46]. Copyright 2016 Elsevier.

Automatic and integrated detection in a microfluidic device was demonstrated by Lee’s group [46,47,56]. They fabricated two layers of PDMS structures and a glass substrate into a device having several chambers and including an external magnet, a micropump and a microvalve. As shown schematically in Figure 5, the experiment started by immobilizing the first aptamer on magnetic beads (MBs) then incubating the target in the micro chamber to form a complex aptamer-MBs-target. The external magnet was used to collect the complex molecules during the washing process, while the unbound and interfering molecules were washed away (Figure 4b step c–d). When the magnetic field was removed, the complex aptamer-MBs-target still remained at the micro-pump. In the next step, the FAM-labeled aptamer was introduced to determine the fluorescent intensity. Taking advantage of another feature of microfluidic design, Dou et al. [48] developed microfluidic droplets-based aptamer-functionalized graphene oxide (GO) to detect low-solubility molecules. The droplet-based design enables the rapid mixing of fluids in the droplet with a high reaction efficiency, even between two different phases of compounds like 17β-estradiol with solvent. The graphene oxide (GO) was used for fluorescence quenching and bonded with aptamer. Their microfluidic device consisted of two layers, the top layer was a PDMS channel with three inlets and one outlet (as the detection zone) and the bottom layer was a glass substrate. The target estradiol was dissolved in ethyl acetate as the oil phase, whereas an aptamer-GO was the aqueous phase. To generate droplets, Dou et al. used a T-junction channel. When the water and the oil phase introduced at different flow rates meet at the T-junction, water-in-oil emulsion droplets will be generated and the aptamer-GO-target complex starts to form at this time. The principle detection of the microfluidic droplets is based on the distance-dependent fluorescence quenching properties of GO. Competitive binding of the aptamer and the target decrease the affinity of the adsorption by GO, this condition may release the aptamer from the GO surface, thus resulting in the fluorescence recovery (“turn-on” of fluorescence intensity). Giuffrida et al. [33] also used microfluidic droplets with a T-junction channel to detect lysozyme. However, their device had six inlets and was equipped with a mixing region and a chaotic mixer channel to allow chemiluminescence detection. The AuNPs was used to enhance chemiluminescence intensity and it was conjugated with the aptamer. Giuffrida et al. reported that their device had several advantages over conventional devices, such as greater sensitivity (femtomolar level), faster detection (10 min), and a low background signal in the absence of the target. Several groups have utilized a microfluidic device for the separation process called microchip electrophoresis (MCE). Lin et al. [41] developed separation techniques on a MCE device based on a tunable aptamer. Different lengths of aptamers could modulate the electrophoretic mobility of proteins and promote effective separation in hydroxyethyl cellulose buffer. Pan et al. [37] proposed laser-induced fluorescence detection (LIF) on a MCE device to detect tumor marker carcinoembryonic antigen (CEA). The application of magnetic beads (MBs) to assist in the target-induced strand cycle would increase the sensitivity.

### 3.2. Paper-Based Microdevice Aptasensors

**Figure 6:**
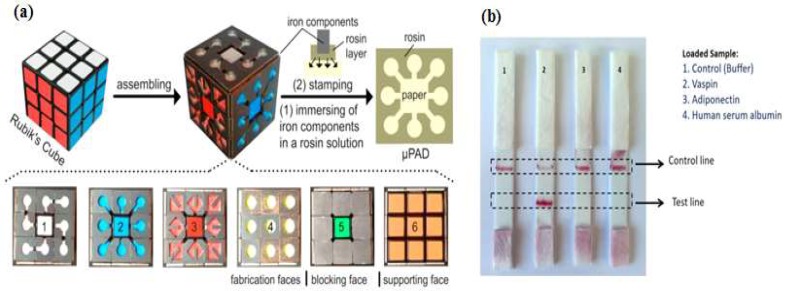
Paper-based analytical device aptasensor. (**a**) Rubik’s cube-based µPAD aptasensor to generate a hydrophobic barrier and a testing zone. Parts 1–5 have different functions while the 6th part acts as a “bare” or support part only. Reproduced with permission from reference [78]. Copyright 2017 Elsevier; (**b**) Lateral strip test for specific detection of vaspin. This device was equipped with a control as indicator. Reproduced with permission from reference [82]. Copyright 2017 Elsevier.

Paper as a substrate in microdevices is a very promising material because its properties provide a versatility of functions. First of all, the cellulose structure allows a passive pump dispenser to be made; the fluid moves by capillary force, which precludes the need for an external instrument. Second, the porous cellulose structure serves to immobilize particles easily. Colorimetry is a common signaling method for obtaining qualitative or semiquantitative results [74]. Since Whitesides’s group revitalized the field of microfluidic paper-based devices in 2007 [75], applications of paper devices have significantly increased due to their simple and low-cost fabrication. Paper-based microdevices can be classified into three main types: microfluidic paper analytical devices, dipstick assays, and lateral flow strip assays [76]. Integrating a paper analytical device and an aptamer to develop sensitive and efficient diagnosis point-of-care-test (POCT) devices for on-site detection was reported by Zhang et al. [77], who developed equipment-free quantitative aptamer-based assays with naked-eye readout to detection adenosine. The super-paramagnetic particles were modified with a short DNA strand for anchoring an aptamer probe. In the presence of the target, the complex aptamer-target was released from the magnetic surface, which then triggered a hybridization chain reaction (HCR) and glucose oxidase was activated to oxidize glucose to H_2_O_2_ and glucose acid. The number of glucose oxidase molecules was proportional to the target concentration. The unique fabrication of a micro paper-based analytical device (µPAD) aptasensor was demonstrated by Fu et al. [78], who were inspired by Rubik’s Cube (RC) toys and formed small iron components to generate hydrophobic barriers through a stamp-mode. The six-faced RCs have different patterns and can be tailored to make multiple combination channels. Fu et al. integrated the portable glucometer readout to detect signals (Figure 6a). During the stamping process, rosin (wax) penetrated into the paper, forming the hydrophobic channel and sample test zone. Although the RC stamp method has good potential for instrument-free sensing, preparing the aptamer sensor, supporting enzyme and carrying out reagent loading remain challenging tasks.

Origami paper analytical devices (oPADs) have been introduced by several groups [79,80,81]. For example, Liu et al. [80] used a glucose oxidase tag to modify the relative concentrations of an electroactive redox couple, and a digital multimeter (DMM) to transduce the result. They folded the chromatography paper into two layers. The first layer, including the sample inlet, was fabricated by wax printing. The second layer was fabricated by screen printing conductive carbon ink. Furthermore, this paper was covered with plastic lamination to prevent fluid evaporation and any contamination. The biotin-labeled aptamer was immobilized on microbeads trapped within the paper fluidic channel and the electrochemical current rise with increasing adenosine concentration. This technique demonstrated a simple preparation when the aptamers immobilized on microbeads. However, the present challenges still occur when the aptamers directly immobilized on the cellulose structure. Yan et al. [79] presented a novel porous Au-paper working electrode on a compatible design origami-electrochemiluminescence (o-ECL). In order to amplify the signal, they used AuNPs, due to their large surface area, stability, and biocompatibility especially with aptamers. The ECL intensity increased only when ATP (adenosine triphosphate) was present. On the other hand, Ma et al. [81] developed the specific recognition of an aptamer and the amplification strategy of a hybridization chain reaction (HCR) using an electrochemiluminescence (ECL) probe (Ru(phen)_3_^2+^). Lateral flow strip assays (LFSAs) are another type of paper-based microdevices. Their simple design allows for on-site detection. Several groups have successfully developed LFSAs combined with aptamer-functionalized AuNPs. As an example, Raston et al. [82] performed an easy fabrication of an LFSAs using a sandwich aptamer conjugated with AuNPs for sensitive vaspin detection. A strip contained three pads: sample pad, nitrocellulose membrane pad, and absorption pad. Two aptamers probes were used that basically functioned as a capturing probe and a signaling probe. When the sample containing vaspin was loaded on a sample pad, the primary aptamer in the test zone captured the vaspin. Thus, the color could be observed in the test zone. For the control experiment, a complementary aptamer in the control zone captured the remaining AuNP-labeled aptamer, thus the signal could always be observed as the control. The signal could only be observed in the presence of vaspin, while no signal was observed in the test zone for adiponectin, HSA (human serum albumin), and buffer as shown in Figure 6b. Wu et al. [83] and Zhou et al. [84] applied this assay strip to get a sensitive and rapid detection of Escherichia coli O157:H7 and Ochratoxin A. They covered the LFSA device with a plastic cover and utilized a portable strip reader to quantify the result. 

## 4. Detection Methods and Assay Formats

### 4.1. Electrochemical Detection Methods

In general, an electrochemical reaction is defined as an electron transfer from a reactant to form a product that gives rise to an electrical current flowing through the cell. Electrochemical detection methods can be divided into three types of dynamic methods. The first type is known as the amperometric method and the current measured at a given electrode potential represents an analytical response that is dependent on the reactant concentration. The second type is known as the voltametric method and the current is measured at a particular potential to obtain good sensitivity and low interference (the current-potential curve is archived for analytical purposes). The third type is called the galvanostatic method and the response is acquired in the form of a potential-time curve. Electrochemical measurements are typically performed using a cell comprised of three electrodes: (1) A working electrode (WE) where the main reaction, such as a redox and immobilization of a probe occur; (2) A reference electrode (RE) that measures the potential of the WE without passing the current through it; and (3) A counter electrode (CE) that serves to set the WE potential and balance current. 

**Figure 7:**
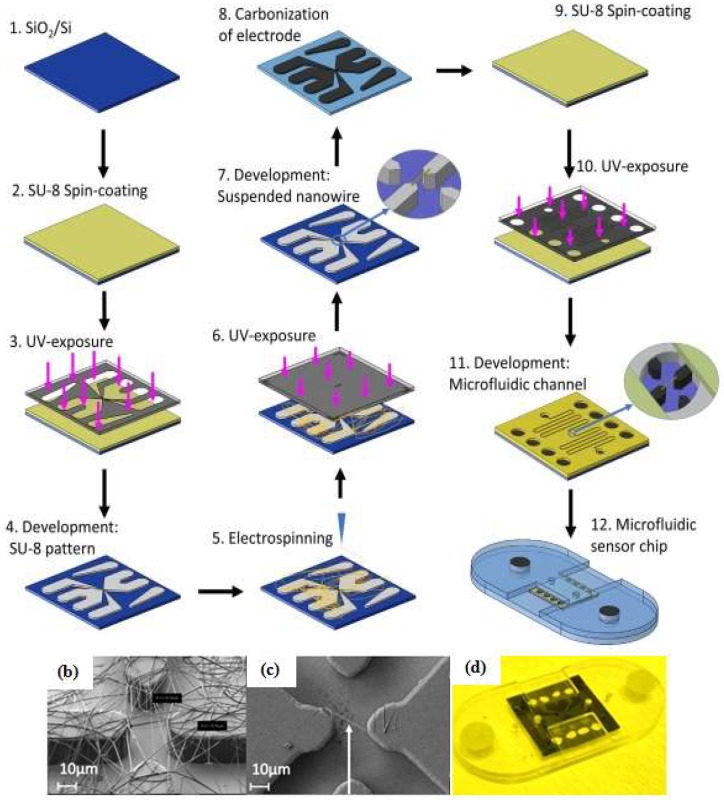
Fabrication steps of the carbon nanowire aptasensor. (**a**) The device was fabricated by integrating electrospinning and photolithography with carbon-microelectromechanical system (C-MEMS) technique; (**b**) Electrospun SU-8 nanowire; (**c**) Single SU-8 nanowire after photolithography and development; (**d**) Microfluidic platform containing the nanowire sensor. Reproduced with permission from reference [72]. Copyright 2018 Elsevier.

Many electrochemical techniques are used in analytical chemistry. The most commonly used ones for microfluidic devices or aptamer biosensors are amperometry [43], voltammetry [31,34,40,44,80,85], and electrochemical impedance spectroscopy [32,35,72,86,87,88,89]. Liu et al. [87] developed ZnO/graphene (ZnO/G) composite with S6 aptamer for a photoelectrochemical (PEC) detector. The AuNPs were electrodeposited on ZnO/G composite that was immobilized with the S6 aptamer, then indium tin oxide (ITO) was used as an electrode to facilitate the ZnO/G composite reaction. As a supporting electrolyte, Liu et al. utilized ascorbic acid as an electron donor for scavenging photogenerated holes under a mild solution medium. The electrochemical impedance spectra were applied to characterize the PEC biosensor and examine each condition (bare, after ZnO/G composite was dropped onto the ITO surface, and the aptamer-target complex form). Sanghavi et al. [40] proposed a unique microfluidic aptasensor that features glassy carbon electrodes and a nanoslit microwells on a glass substrate. Their method does not require a labeling, immobilizing, or a washing process. Aptamer-functionalized AuNPs were used to enhance the net area available for target cortisol capture and to enable the unhindered diffusion of analytes towards the binding surface. Square wave voltammetry (SWV) data were acquired by scanning the potential of the working electrode toward the positive direction in the −0.5 to −1.2 V range with frequency 100 Hz. Another electrochemical technique was developed by Chad et al. [66]. They proposed a microfluidic electrolyte-insulator-semiconductor (EIS) chip based on ion-sensitive field-effect transistor with capacitive detection. The working principle of the proposed device is the change of the gate voltage that occurs due to the release of protons or intrinsic charge biomolecules during biomolecule interactions. A thiolated aptameric peptide was immobilized on AuNPs for recognition of a protein kinase A (PKA) target. Interaction between the aptamer and target led to a shift in the gate voltage. Recently, Thiha et al. [72] presented a fabrication technique for a suspended carbon nanowire sensor (sub-100 nm diameters) by simple electrospinning and applying carbon-microelectromechanical system (C-MEMS) techniques (Figure 7). The C-MEMS techniques provided patterning of the polymer (typically SU-8 photoresist) with a high aspect ratio and 3D structures shape. After the patterning process, the polymer was pyrolyzed and electrospun to obtain carbon nanostructures, then it was integrated with a microfluidic chip to form a label-free chemiresistive biosensor. The amine-functionalized aptamer was covalently attached to carboxylic groups with the assistance of sulfo-*N*-hydroxysuccinimide (sulfo-NHS) and *N*-(3-dimethylamnopropyl)-*N*-ethylcarbodiimide hydrochloride (EDC). The detection principle is based on conductivity changes that occur when the target binds on the suspended nanowire. The current-potential (I-V) was characterized before and after incubating with the target and the resistance value (R) was obtained from the inverse of the I-V curve slope. The percent ratio change of the resistance was calculated as ΔR/R_0_, where ΔR is the difference in resistance after incubation with target (R) and the original resistance (R_0_).

### 4.2. Optical Detection Methods

The analytical techniques based on light interaction with a sample are known as optical detection methods. To obtain an optical sensor, a specific reagent is involved in a sensing layer and its reaction process is monitored by a light beam that is conveyed by optical fibers. An optical transducer was obtained after measuring the absorbed or emitted light power on the sensing layer. As the dependence of light power on the wavelength represents an optical spectrum, consequently the application of this method needs a component that is able to absorb or emit light. Otherwise, some external molecule may be used as an optical label. Fluorescent materials [13,17,19,21,28,37,38,42,45,46,47,48,50,53,54,55,56,60,61,68,69,90,91,92,93,94,95,96], and dyes (colorimetry) [25,64,74,77,78,82,83,84,97,98] are commonly used as labels in microdevices based on aptasensors.

#### 4.2.1. Fluorescence Methods

**Figure 8:**
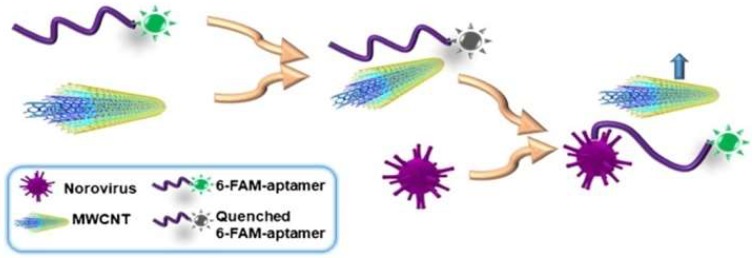
Schematic illustration of “signal-on” aptasensor based on MWCNT and fluorescence-labeled aptamer. Reproduced with permission from reference [92]. Copyright 2017 Springer.

Florescence methods consist of light emission by molecules previously excited through light absorption. Weng and Neethirajan [92] used 6-carboxyfluorescein (6-FAM) as the aptamer label and multi-walled carbon nanotubes (MWCNTs) or graphene oxide (GO) for the quencher in their device. When the target norovirus was present, fluorescence was recovered due to the release of the labeled-aptamer from the MWCNT surface and it was detected at Ex/Em = 490 nm/520 nm by the multi-mode reader Figure 8. The “signal-on” fluorescence aptasensor was also demonstrated by Ueno et al. [55]. They demonstrated a portable design with a multichannel chip for simultaneous detection of three to five samples. A recent update on a fluorescence aptasensor was presented by Jin et al. [95]. This group developed nanocomposites composed of magnetic Fe_3_O_4_-aptamer-carbon dots that exhibited down-conversion fluorescence (DCF) and up-conversion fluorescence (UCF) emissions simultaneously. The UCF emission wavelength is shorter than its corresponding excitation wavelength, whereas the DCF (usually called fluorescence) is the opposite. The high binding affinity between the target and aptamer could induce unwinding of the carbon dots from the target-aptamer complex and recovery of the UCF signal. Therefore, in the presence of the target, the UCF signal (peak at 475 nm) gradually increased.

#### 4.2.2. Colorimetry Methods

**Figure 9:**
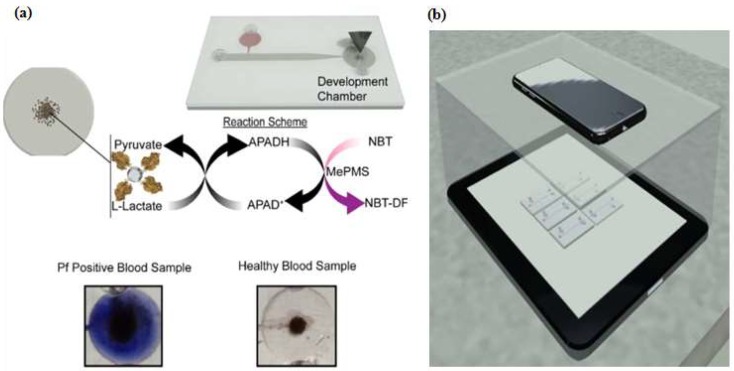
Microfluidic Aptamer-Tethered Enzyme Capture (APTEC) biosensor. (**a**) The reaction scheme of the reagents and redox reaction that results in the generation of an insoluble purple diformazan dye. There was a color difference between positive and negative samples; (**b**) The smartphone camera was used for capturing images in a telemedicine application. Reproduced with permission from reference [97]. Copyright 2018 Elsevier.

Colorimetry methods are commonly used to determine the concentration of a solution by measuring the absorbance of at a specific wavelength, this approach is also applied in lateral strip detection with a control or known concentration [82,83,84]. Simple to enable and develop, instrument-free colorimetry is favorable. Wei et al. [98] and Zhang et al. [77] developed instrument-free detections using a microfluidic aptasensor: the colored result could be identified easily by the naked eye. Another advantage of a colorimetry-integrated microdevice was utilized by Fraser et al. [97]. They designed an integrated Aptamer-Tethered Enzyme Capture (APTEC) on a microfluidic device and applied it for a telemedicine application. The APTEC technique has three main steps: First, micromagnetic beads (µMBs) were coated with the aptamer via a streptavidin-biotin interaction. Then the coated beads were incubated on lysed sample of human blood. When the target was present, the aptamer-coated µMBs bound specifically to the target (protein PfLDH). Second, the unbound molecules and other contaminants were washed and removed by the mobile phase. Third, the aptamer-coated µMBs-target was transferred by mobile phase to the development chamber that contained the development reagent and a stronger colorimetry signal was generated. The non-target sample would not develop a colorimetry signal in the described assay (Figure 9). For signal analysis, the microdevice was placed on the top of an iPad that displayed a homogenous white light then covered with an opaque box. The smartphone camera was used for capturing the images and coupled with supporting information such as time, date, and GPS coordinates for the telemedicine application. Furthermore, the receiver analyzed the images with ImageJ software.

### 4.3. Miscellaneous Methods

#### 4.3.1. Surface Plasmon Resonance (SPR) Methods

Large groups of electrons in an oscillating state form a surface of plasmons, which is a phenomenon known as SPR. The SPR depends on three factors: angle of incident, wavelength of the radiation, and refraction index of the sample. These methods are routinely used for investigating molecular interactions. Dausse et al. [23] demonstrated an SPR method for sequence selection during the SELEX method, called SPR-SELEX, that could perform selection and evaluation simultaneously. Other groups utilized a microfluidic aptasensor integrated with an SPR sensor to realize rapid and easy-to-use quantitative analysis [26,99].

#### 4.3.2. Surface Acoustic Wave (SAW) Methods

**Figure 10:**
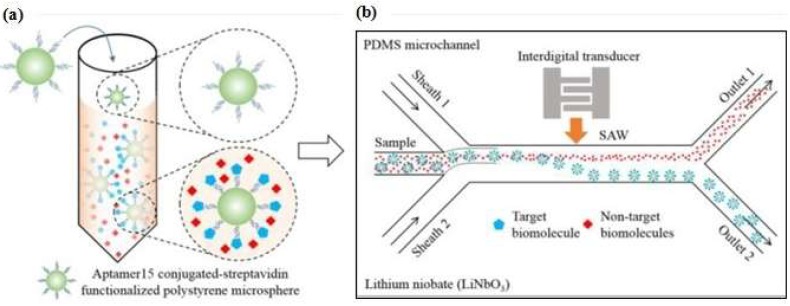
(**a**) Specific aptamer to form a microparticle−aptamer−target complex; the unbound particles remained in a free condition; (**b**) Separation process of the mixture solution through an acoustofluidic device. Reproduced with permission from reference [100]. Copyright 2017 American Chemical Society.

These methods are based on acoustic excitation by means of two electrodes placed on the same surface interdigitated transducer (IDT) configuration. The acoustic wave induced by an IDT is propagated in a thin layer at a piezoelectric surface. Ahmad et al. [100] proposed a microfluidic device that applies acoustic waves to drive functionalized microparticles into a continuous flow microchannel to separate particle-conjugated target proteins from the sample. This platform utilized an IDT transducer (with an Au-Cr layer) that was patterned on top of the piezoelectric lithium niobate (LiNbO_3_) substrate to generate high-frequency surface acoustic waves (SAWs). The aptamer was conjugated to streptavidin-functionalized polystyrene microparticles and incubated with a sample mixture. When the target thrombin was present, the aptamer formed a microparticle-aptamer-target complex and other molecules remained in a free condition. Once the high-frequency SAWs were actuated, the complex aptamer was separated from the mixture due to the lateral migration of fluid under the influence of the acoustic radiation force and collected in outlet 2 (Figure 10). Furthermore, Zhang et al. [101] proposed a microfluidic love-wave sensor that is a special type of SAW sensor that uses a shear horizontal wave to reduce energy dissipation and to increase the surface sensitivity. The device was prepared on a LiTaO_3_ (lithium tantalate) substrate with an aluminum IDT and functionalized with aptamer.

#### 4.3.3. Chemiluminescence and Electrochemiluminescence Methods

Luminescence, as a general term is related to the energy transition between molecular orbitals that produces an emission of light. When the excitation of the molecules is caused by a chemical reaction, this light emission is chemiluminescence [27,33,36,58,71,81]. The emission that accompanies an electrochemical reaction is known as electrochemiluminescence [79]. Costantini et al. [58] developed an aptamer-linked immobilized sorbent assay (ALISA) that was performed in a microfluidic device that had a functionalized poly(2-hydroxyethyle methacrylate) PHEMA polymer brush layer on a glass substrate. The ALISA relied on the formation of a sandwich-like structure consisting of the target and two target related-aptamers. The first aptamer was bounded on PHEMA to capture the target and the other aptamer was a biotin-labeled probe. The avidin-labeled HRP (horseradish peroxidase) would give a chemiluminescent signal after binding with the biotin, this signal indicated that PHEMA-aptamer was interacting with the target.

## 5. Target Analytes

### 5.1. Disease Markers

As described in Section 4, microfluidic aptasensors have numerous advantages for point-of-care detection, mostly as disease markers. Thrombin is a critical biomarker for Alzheimer’s disease and it is a well-known target for a microfluidic aptasensor and every year several researchers have reported updates for thrombin detection that offer more sensitivity. Lin et al. [64] proposed a very sensitive detection of thrombin from human plasma serum with a detection limit 0.082 pg·mL^−1^ and a linear range 0.1–50.000 pg·mL^−1^. On the other hand, some groups focused on improving the detection method. For example, Zhao et al. [25] developed a microfluidic chip without signal amplification and using only naked-eye detection. The detection limit was 20 pM, this result is quite satisfying for simple detection purposes. Song et al. [60] used a sandwich aptamer-target-aptamer to assay thrombin with high selective detection even in the presence of concentrated bovine serum albumin (BSA). They obtained a thrombin detection limit of 25 pM. Uddin et al. [29] used a device with attractive disk and microbeads to reduce the sample-to-result time from 40 min to 15 min while using only 10 µL of sample volume. They obtained a thrombin detection limit of 25 pM.

### 5.2. Viruses and Bacteria

The detection of viruses and bacteria in real samples is important for dealing with environmental contamination or foodborne diseases. Commonly, their detection relies on culture-based tests, antibody-based tests, and polymerase chain reaction (PCR)-based tests. Despite their usefulness, these methods are costly and time-consuming. Neethiarajan group’s [31,92] successfully developed a simple microfluidic aptasensor for norovirus detection with low detection limits (100 pM). The device not only had good sensitivity, but was also selective to norovirus even in the present of interferon. Moreover, the total analysis time was significantly reduced compared with the conventional method. Wang et al. [46] demonstrated a fluorescent-labeled universal aptamer to determine three different influenza viruses (influenza A-H1N1, H3N2, and influenza B) at the same time in 20 min. Another multiple detection was developed by Zuo et al. [90]. Their microdevice was able to detect multiple bacteria (Lactobacillus acidophilus, Staphylococcus aureus, Salmonella enterica) at the same time. This device was consisted of a ready-to-use microfluidic aptasensor with a detection limit of 11.0 CFU·mL^−1^ and total time for detection was about 10 min.

### 5.3. Antibiotics

Antibiotic residues in foodstuffs pose certain hazards to human health among people who are sensitive to antibiotics, have an imbalance of intestinal microbiota or have bacterial resistance. Unfortunately, many of these residues are unintentionally consumed because some of the conventional methods may not meet the need for fast and high throughput analysis in food safety screening. Recently, the detection of multiple antibiotic residues based on a microfluidic aptasensor has been developed to fulfill these needs in food safety screening. The detection principle is based on microchip electrophoresis (MCE) and the target is a catalyzed hairpin assembly. The device could simultaneously detect kanamycin and oxytetracycline with detection limits of 0.7 pg·mL^−1^ and 0.9 pg·mL^−1^, respectively [102]. Using a similar MCE method, Zhou et al. [103] developed a label-free and sensitive detection of chloramphenicol that reached a detection limit of 0.003 ng·mL^−1^. Hou et al. [86] reported the fast detection of tetracycline using an interdigital array microelectrode (IDAM). The IDAM was integrated with impedance detection into a miniaturized conventional electrode and it was able to detect 1 nM of tetracycline in a milk sample.

### 5.4. Toxins

A rapid, sensitive, and specific assay technique was developed for routine analysis in foods and animal feedstuffs. Several researchers proposed a microfluidic aptasensor assay to analyze mycotoxin [50,58,62]. A lateral flow strip aptasensor assay was developed to detect ochratoxin A more easily. To perform a test, only the minimum sample volume and reagent volume were needed. The whole process was completed within 15 mins and a visual detection limit of 1 ng·mL^−1^ was obtained [84]. This assay was suitable for rapid and on-site detection, especially for screening raw materials in the animal feed production industry. Another challenging factor to analyze these toxins is isolation from the real samples. The uneven distribution of mycotoxin in matrix samples should be considered to apply additional steps on sample preparation. In recent years, marine toxins have drawn the attention of scientists due to the increased consumption of sea products. Certain toxins have been identified: saxitoxins, tetrodotoxin, okadaic acid, brevetoxins, and gonyautoxin ¼. Although these toxins are mostly produced by microalgae, especially dinoflagellates, it is now clear that bacteria are responsible for the production of some toxins. Handy et al. [24] published the first article related to marine toxin detection with an aptasensor, specifically saxitoxin. They developed saxitoxin-aptamer sequences by the SELEX method and evaluated the binding affinity with the SPR method. Tetrodotoxin is one famous marine toxin because of its involvement in the fatal food poisoning found in puffer fish, starfish, and blue-ringed octopus. Recently, a sensitive detection of tetrodotoxin using a microfluidic aptasensor was developed by Jin et al. [95], with a detection limit of 0.06 ng·mL^−1^. Okadaic acid is known as diarrhetic shellfish toxin (DST) and is found in contaminated shellfish. Various microfluidic techniques for okadaic acid detection have been developed, including interdigitated microelectrodes with AuNPs [73], a paper-based aptasensor [61], and an enzyme-linked aptamer assay (ELAA) [22]. In the ELAA competitive assay, the lowest limit of detection reached 0.01 ng·mL^−1^ and the widest detection range was from 0.025 to 10 ng·mL^−1^ in spiked clam samples. The binding affinity of an aptamer to detect brevetoxins and gonyautoxin-1/4 has been tested. The lowest dissociation constants for brevetoxin were 4.83 µM [104] and for gonyautoxin ¼ 17.7 nM [105].

## 6. Conclusions and Future Perspectives

Applications of aptasensors on microdevices have led to positive outcomes in bioanalysis. This paper has attempted to offer readers an overview of recent trends and advancements in the development and application of microdevices based on aptamer sensors. Table A1 (Appendix A) summarizes device features, including their classifications and assay formats. Microdevice sensors in flow analysis systems deals with the control and manipulation of fluid volumes in the submicroliter region that are constrained to very small size channels. The fluid flow can be prompted by applied pressure or electrokinetics. What distinguishes microdevice systems from a conventional flow analysis systems is the integration of a large network of channels and other microdevices (such as actuators and valves) on a small chip. The major concepts and principles of device fabrication still rely on photolithography, etching, bonding, screen printing, doping, and thin film formation. These fabrication techniques give rise to various collaborations in multidisciplinary research. The utilization of new nanomaterials (metal nanoparticles, polymer nanoparticles, carbon dots, magnetic beads, and micro beads) has promoted the development of aptamer sensors that offer high throughput and good sensitivity. Many innovations presented in the literature are still at the proof-of-concept state. However, some have already been applied to commercial applications, such as the lateral flow strip assay. This technique does not require a sophisticated instrument or may even be instrument-free as a result of naked-eye detection.

Based on the current circumstances in the field of bioanalysis, several points that can be considered in the future are noted: (1) Despite their many advantages over other conventional methods, the scaling down of existing procedures to use microdevice-based aptasensors sometimes needs to be improved from the onset; (2) The simplest design is not always related to the smallest dimension. The movement towards ergonomic design, easy to handle, and cost-effective devices will certainly occur; (3) Marine toxins have attracted attention due to the increased human consumption of marine products. However, detections using microfluidic-based aptasensors are still limited to only a few toxins. The continued developments of such methods are expected in the near future.

Developing relatively simple and sensitive microdevices that are easily fabricated and combining them with automatic and embedded elements in compatible substrates by micro-total analytical systems (µTAS) will certainly increase in the coming years.

## Appendix A

**Table A1:** Summary of microdevice-based aptasensors on several platforms and target analytes.

Detection Method	Substrate	Aptamer	Target	Matrix Sample	LOD or Linear Range	Device Features	Reference
*Electrochemical*							
Chronoamperometry	Glass	Peptide	Thrombin	-	10 fg·mL^−1^ to 1 μg·mL^−1^	Plasma-functionalized SWCNT	[43]
DPV	PDMS	Biotin-Aptamer-Ferrocene	Norovirus	Bovine Blood	100 pM 100 pM to 3.5 nM	Integrated PDMS-SPCE Graphene-Au composite Switch-off signal	[31]
SWV	Glass	Competitive aptamer	Cortisol	Saliva glucocorticoids in serum	10 pg·mL^−1^ 30 pg·mL^−1^ to 10 µg·mL^−1^	Sample volume (<1 μL) Graphene modified electrode	[40]
SWV	Glass	MB-labeled Aptamer	TGF-β1	Human hepatic stellate cell	1 ppb	PDMS layer with microcup Comparing with ELISA	[44]
Digital multimeter	Chromatography paper	-	Adenosine	-	11.8 µM	Origami paper device Attractive design	[80]
DPV	Paper	Peptide	Renin	-	300 ng·mL^−1^	DEP (disposable electrochemical printed) Uses SPR to check binding affinity	[85]
EIS	Poly-imide film	-	Bisphenol A (BPA)	Food (canned)	152.93 aM 1 fM to 10 pM	Printed circuit board material Rapid detection (20 s)	[32]
EIS	Glass	-	Avian Influenza Virus	Virus culture	0.0128 hemagglutinin units (HAU)	Interdigitated electrode On site detection SELEX on Chip	[35]
Resistance	Si-Wafer	Amine-functionalized aptamer	Salmonella typhimurium	Fresh beef	10 CFU·mL^−1^	Carbon nanowire sensors C-MEMS Rapid detection (5 min)	[72]
EIS	Glass	-	Tetracycline	Milk	1 pM	Multi-walled carbon nanotubes Interdigital array microelectrode	[86]
Photoelectrochemical	Indium Tin Oxide (ITO)	S6 aptamer	SK-BR-3	-	58 cell·mL^−1^ 10^2^ to 10^6^ cells·mL^−1^	ITO-based SPEs device Disposable ITO device	[87]
EIS	Cyclic olefin copolymer	Short strand aptamer	Ampicillin Kanamycin A	UHT low fatm milk	10 pM A = 100 pM to 1 mM K = 10 nM to 1 mM	PEDOT-OH:TsO All polymer substrate	[88]
Detection Method	Substrate	Aptamer	Target	Matrix Sample	LOD or Linear Range	Device Features	Reference
EIS	Glass	Sgc8 TD05	CCRF-CEM Ramos cells	T-cell acute lymphoblastic leukemia (ALL)	-	Logic aptamer sensor (LAS) Simple detection with digital multimeter	[89]
*Optical*							
Fluorescence	Glass	Aptamer-antibody sandwich	Cancer stem-like cells	-	-	Cell-SELEX Automatic device Heater—cooling chip	[13]
Fluorescence	Glass	Aptamer sandwich with magnetic beads	Human immunoglobulin A (IgA)	Random oligonucleotides	-	Microfludic SELEX Fully integrated platform	[17]
Fluorescence	Glass	-	Malaria parasite	Red blood cells	-	I-SELEX Only requires syringe pump	[19]
Fluorescence	Glass	-	-	Mixed cells	-	Cell-SELEX Dielectrophoresis and electrophoresis	[21]
Fluorescence	PDMS	Hair pin aptamer	Protein tyrosine kinase-7	Cell culture	0.4 nM	Laser-induced fluorescence detector (LIFD) Microfluidic droplet	[28]
Fluorescence	Glass	FAM-aptamer	Carcinoembryonic antigen (CEA)	Human serum	68 ng·mL^−1^ 130 pg·mL^−1^ to 8 ng·mL^−1^	Micro chip electrophoresis (MCE)	[37]
Fluorescence	Glass	Cy3-aptamer	Thrombin	Human serum	0.4 fM	Avidin-biotin interaction Use 2 kinds of aptamer	[38]
Fluorescence	Glass	Photoluminescent GOQD-aptamer	Lead ion (Pb^2+^)	Drinking water Tap water Lake water	0.64 nM 1 to 1000 nM	Packed with cation exchange resins Peristaltic PDMS Micropump	[42]
Fluorescence	Glass	G-quadruplex	VEGF-165 protein	DMEM cell media	0.17 pM 0.52 to 52.00 pM	Label-free In the presence of Ir(III) no signal	[45]
Fluorescence	Glass	FAM-aptamer universal	Influenza virus	Random oligonucleotides	3.2 HAU	Automatic process Rapid detection	[46]
Fluorescence	Glass	FAM-aptamer sandwich	Influenza A (InfA/H1N1)		0.032 HAU	Magnet external Rapid detection	[47]
Fluorescence	Glass	Fluorescence-labeled	17β-estradiol	Estradiol solution	0.07 pM	Microfluidic droplet Turn-on signal	[48]
Fluorescence	Glass	G-quadruplex structure	Ochratoxin A	-	-	Fluorescence polarization	[50]
Fluorescence	Glass	Multivalent DNA aptamer nanospheres	Human acute leukemia cells	Human blood	-	Flow cytometry analysis Rapid detection	[53]
Detection Method	Substrate	Aptamer	Target	Matrix Sample	LOD or Linear Range	Device Features	Reference
Fluorescence	Glass	FAM-aptamer	Thrombin Prostate specific antigen (PSA)	-	-	FRET Longer spacer gives good sensitivity	[54]
Fluorescence	Glass	FAM-aptamer	Thrombin Prostate specific antigen (PSA) Hemagglutinin	-	-	FRET Multiple target Aptamer immobilize on GO flakes	[55]
Fluorescence	Glass	Sandwich aptamer FITC	Glycated hemoglobins (HbA1c) & Total hemoglobin (Hb)	Blood	-	Automated microfluidic system Low reagent consumption	[56]
Fluorescence	Glass	Sandwich aptamer	Thrombin	-	27 pM	Gold nanohole array Nanoimprinting technology	[60]
Fluorescence	Glass	Aptamer functionalize QD	Lysozyme, OA, Brevetoxin, ß-conglutin lupine	Fresh egg white Mussel tissue Sausage	Lysozyme (343 ppb); OA (0.4 ppb); Brevetoxin (0.56 ppb); ß-cl(2.5 ppb)	Quantum Dots (QD) GO-quencher Comparing with ELISA	[61]
Fluorescence	Si-nanowire	Cocktail aptamer	Non-small cell lung cancer	Blood	-	PDMS chaotic mixer Aptamer grafted Si-nano wire substrate	[68]
Fluorescence	Glass	FAM-aptamer	ss-DNA	-	-	Isolating ssDNA from dsDNA PC membrane	[69]
Fluorescence	Chromatography paper	Aptamer-functionalized GO	Staphylococcus aureus	Buffer (Bacterial colonies)	11.0 CFU·mL^−1^	PDMS/paper/glass microfludic device Fast detection	[90]
Fluorescence	Paper	-	Cancer cells	Cell culture	MCF-7: 6270 cell·mL^−1^ HL-60: 65 cell·mL^−1^	Mesoporous silica nanoparticles (MSNs) Naked-eye detection	[91]
Fluorescence	Paper	FAM-aptamer	Norovirus	Spiked mussel sample	MWCNT: 4.4 ng·mL^−1^ GO: 3.3 ng·mL^−1^ 13 ngmL^−1^ to 13 µg·mL^−1^	Multi-walled carbon nanotubes Graphene oxide	[92]
Fluorescence	Printed circuit board (PCB)	-	Cocaine Adenosine	Human blood serum	Cocaine: 0.1 pM Adenosine: 0.5	MECAS-chip Simultaneous detection	[93]
Fluorescence	Glass	FAM-aptamer	Lysozyme	-	-	Electrophoresis frontal mode FACME method	[94]
Fluorescence	-	Amine-aptamer	Tetrodotoxin (TTX)	Human blood Urine	0.06 ng·mL^−1^ 0.1 ng·mL^−1^ to mg·mL^−1^	Marine toxin Fe_3_O_4_/apt/CD composite	[95]
Detection Method	Substrate	Aptamer	Target	Matrix Sample	LOD or Linear Range	Device Features	Reference
*Colorimetry*							
Colorimetry	Glass	Sandwich aptamer	Thrombin	-	20 pM	Naked-eye & Flatbed detection Micro pump	[25]
Colorimetry	Si-wafer	G-quadruplex structure	Thrombin	Human blood	0.083 pg·mL^−1^ 0.1 to 50.000 pg·mL^−1^	Rolling circle amplification Micro channel	[64]
Colorimetry	Paper	Cross-linking aptamer	Cocaine	Urine	7.3 µM	Utilizes ImageJ software Hydrogel-µPAD	[74]
Colorimetry	Paper	Hybridization chain reaction	Adenosine	Human serum	1.5 µM 1.5 µM to 19.3 mM	Naked eyes detection Uses superparamagnetism	[77]
Colorimetry	Paper	Aptamer attached microbeads	Adenosine	Urine	-	Rubik’s cube stamp Stamping method	[78]
Colorimetry	Paper Cellulose fiber	Sandwich aptamer	Vaspin	Buffer & serum	Buffer: 0.137 nM Serum: 0.105 nM	Lateral strip assay Naked-eye detection	[82]
Colorimetry	Paper Cellulose fiber	Biotin modified aptamer	*E. coli* O157: H7	Culture E.coli	10 CFU·mL^−1^	Lateral strip assay Naked-eye detection	[83]
Colorimetry	Paper Cellulose fiber	Competitive aptamer	Ochratoxin A	-	1 ppb	Lateral strip assay Naked-eye detection Rapid detection	[84]
Colorimetry	Clear resin	Biotinylated aptamer	PfLDH enzyme (Malaria)	Human blood serum	0.01%	Telemedicine Ipad - Iphone detection 3D printing resin	[97]
Colorimetry	Paper	Hydrogel-aptamer	Cocaine Adenosine Pt ^+2^	Urine	-	Naked-eye detection Signal off-on by interaction apt-target	[98]
*Miscellaneous*							
Surface Plasmon Resonance		Hairpin RNA aptamer	Aptamer candidate	Random library	K_D_ = 8 nM	SPR-SELEX SELEX on chip	[23]
Surface Acoustic Wave	PDMS	Polystyrene aptamer conjugate	Thrombin	Buffer	-	Acoustic wave driven Interdigitated transducer	[100]
Surface Acoustic Wave	LiTaO_3_ substrate with SiO_2_ film	Aptamer beacon	Prostate specific antigen (PSA) ATP	-	PSA = 10 ppb 10 ppb to 1 ppm ATP = 0.1 pM 0.5 pM to 7 nM	Interdigitated transducer Utilized AuNPs	[101]
Chemiluminescence	PDMS	Aptamer-antibody sandwich	free prostate specific antigen (fPSA)	Human semen	0.5 ng·mL^−1^	Performed in parallel Antibody labeled HRP	[27]
Chemiluminescence	PDMS	Thiolated aptamer	Lysozyme	Human serum	44.6 fM	Droplet microfluidic Digital microfluidic Low sample volume	[33]
Detection Method	Substrate	Aptamer	Target	Matrix Sample	LOD or Linear Range	Device Features	Reference
Chemiluminescence	Glass	Aptamer-antibody sandwich	HbA1c	Blood	0.65 g·dL^−1^	Three-layer chips Detection time 25 min Utilizes magnetic beads	[36]
Chemiluminescence	Glass	-	Ochratoxin A	Beer	0.82 mg·L^−1^	Polymer brush ALISA	[58]
Electrochemiluminescence	Paper	Sandwich aptamer	ATP	-	0.1 pM 0.5 pM to 7 nM	Origami design Modified porous paper	[79]
